# What Causes Health Promotion Behaviors in College Students?

**DOI:** 10.2174/1874434601812010106

**Published:** 2018-06-29

**Authors:** Mi Young Kim, Yu Jeong Kim

**Affiliations:** 1 Departnment College of Nursing, Eulji University, Seongnam, South Korea; 2 Department of Nursing, Hoseo University, Asan, South Korea

**Keywords:** Health Behavior, Health Cognition, Health Perception, Self-Concept, Young Adult

## Abstract

**Background:**

College students are exposed to an environment that places their health at risk. As a result, they are vulnerable to health-related problems. In order to improve health promotion behaviors, it is necessary to identify the variables affecting these behaviors. However, few studies have comprehensively examined health consciousness, health perception, and self-esteem as variables in health promotion behaviors among college students.

**Objective:**

The purpose of this study is to identify the factors influencing health promotion behaviors in college students.

**Method:**

Data were collected from 331 students, using a structured questionnaire based on the Health Promoting Lifestyle Profile (health promotion behaviors), Dutta-Bergman’s Health Consciousness Scale (health consciousness), the Health Perception Questionnaire (health perception), Rosenberg’s Self-Esteem Scale (self-esteem), and sociodemographic data. To assess the factors that influence health promotion behaviors, a multiple regression analysis was performed.

**Results:**

Health promotion behaviors were higher when health cognition was higher (r=.421, *p*<.001), health perception was higher (r=.326, *p*<.001), and self-esteem was higher (r=.526, *p*<.001). The constructed model for health promotion behaviors showed that the statistically significant explanatory variables were health cognition, health perception, and self-esteem. The model explained 34.9% of the variance in health promotion behaviors.

**Conclusion:**

It will be necessary to develop an intervention program targeting health cognition, health perception, and self-esteem in order to increase health promotion behaviors in college students.

## 1 INTRODUCTION

### 1.1 Background

As a major variable influencing individual well-being, health-related habits, especially those established during college, continue to affect health after early adulthood [1]. Nevertheless, college students experience a lack of sleep and psychological stress due to an irregular lifestyle filled with various scholastic events and gatherings - including festivals and outings - alongside a heavy academic workload and job preparation activities. They are also easily exposed to smoking and drinking. Such an environment has a negative effect on their physical and mental health [2]. As such, the college period is one in which students are exposed to an environment that places their health at risk, although the threat to their health is not necessarily obvious. This period is regarded as one in which students are vulnerable to health-related problems because activities promoting health are not readily available. This vulnerability is compounded by the lack of concern of students to health management [3], and the ease with which the importance of health is ignored [2].

Health promotion is an educational, social, and environmental approach intended to improve health through the introduction of beneficial lifestyles and habits. It is designed to help people reach optimal levels of physical, mental, social, and spiritual well-being [4]. In other words, health promotion behaviors involve performing proactive activities for self-actualization and improvement, as well as for disease treatment and prevention [5]. In the US, efforts are being made to educate college students about the importance of health management and how lifestyle changes can be made accordingly [6]. Health management in Korea, however, focuses on middle- and late-adulthood because these are the periods in which health-related problems become apparent. In Korea, there is relatively less interest in health-related problems of college students [7]; indeed, health promotion tends to decrease sharply after the age of 18, which is the age at which most people start college [8]. It is very important to establish health promotion among college-age students because it is relatively easier to change behavioral patterns during early adulthood, rather than in middle- and late adulthood. Moreover, habits formed during this period can determine later patterns of health behavior [3]. Thus, an effort to improve health promotion behaviors among college students is necessary. In order to achieve this, it is first necessary to identify the variables affecting these behaviors.

According to previous studies, variables influencing health promotion and behavior include sex, religion, type of residence [9], and university year [10]. Key cognitive variables include health consciousness-that is, how one thinks about one’s health status [11]-and health beliefs, which are how an individual’s subjective beliefs shape their behavior related to preventing disease [12]. In addition, health perception-a subjective process of how health-related issues are understood-is presented as a variable affecting health promotion behaviors [7]. There are also several socio-psychological variables including socio-psychological health [10], self-efficacy, perceived benefit and disability, health status and importance [13], self-esteem, and social support [14]. Among these, low self-esteem contributes to disease, as low self-esteem decreases belief in the ability to influence one’s environment and situation, which in turn affects health promotion behaviors [15]. This could be an important variable in the promotion of health behavior among college students, who enter an autonomous environment, and experience dramatic physical, emotional, and social changes as they transition into adulthood. Nonetheless, there are few studies that comprehensively examine health consciousness, health perception, and self-esteem as variables affecting health promotion behaviors among college students. This research focuses on the impact that attitudes towards health have on health promotion in college students; the factors considered include health consciousness and health perception (*i.e*., subjective understanding of one’s health), as well as self-esteem (*i.e*., attitude towards oneself). Accordingly, this study investigates the levels of health consciousness, health perception, self-esteem, and the promotion of health behaviors among college students. In doing so, this research identifies the variables affecting health promotion and behavior, and provides baseline data for the development of interventions in health promotion activities for college students.

### 1.2 Research Purposes

The purpose of this study is to examine the levels of health consciousness in college students; it examines their health perception, self-esteem, and health promotion behaviors, in order to identify differences based on general characteristics. In doing so, this study will identify the variables that influence health promotion behaviors. The specific purposes of this research are summarized as follows:

First, this research aims to identify the degree of health consciousness in college students, as well as their health perception, self-esteem, and health promotion behaviors.

Second, this research aims to identify differences in health consciousness, health perception, self-esteem, and health promotion behaviors depending on general characteristics.

Third, this research aims to identify possible correlations between health consciousness, health perception, self-esteem, and health promotion behaviors.

Fourth, this research aims to identify variables that predict the promotion of health behavior among college students.

## 2 MATERIALS AND METHODS

### 2.1 Research Design

This is a quantitative cross-sectional descriptive survey intended to identify the level of health promotion behaviors among college students and the related variables.

### 2.2 Research Participants

The participants of this research were students from two universities in Korea. The required sample size was calculated using G*Power 3.1 [16] with an effect size of .04, a power of .8, an α of .05, and the number of predictors as 3; this produced the result of 277 participants. Based on this number, this study took a dropout rate of 20% into consideration, hardcopy questionnaires were disseminated to 335 people.

### 2.3 Research Tools

#### 2.3.1 Health Consciousness

Health consciousness is a concept indicating how integrated one’s interest in health is with one’s daily activities [17]. This research used a tool adapted by Suh [18] for the Korean context from Dutta-Bergman’s health consciousness scale [19], to measure college students’ health consciousness. Using a 5-point Likert scale, this tool has five questions with “strongly disagree” as a 1-point response and “strongly agree” as a 5-point response. Higher points indicate a higher level of health consciousness, which suggests an individual is health-oriented and has a positive attitude towards behaviors such as exercise and eating healthy food. In terms of the tool’s reliability, Cronbach’s α was .80 in a previous study [18], and .79 in this research.

#### 2.3.2 Health Perception

Health perception refers to self-evaluation of an individual’s physical and mental well-being [20]. This research used a tool adapted from the Health Perception Questionnaire developed by Ware [21], which was revised accordingly for the Korean context and assessed for adequate reliability and validity [22]. This tool consists of six categories: current health, past health, future health, health interest and concern, resistance and sensitivity, and patient role rejection. This tool has 20 questions measured on a 5-point Likert scale, with “never” as a 1-point response and “always” as a 5-point response, with the final score ranging between 20 and 100 points. Negative questions were inversely scored, such that higher scores on this scale indicated a higher degree of health perception. In terms of the tool’s reliability, Cronbach’s α was .91 in a previous study [23], and .74 in this research.

#### 2.3.3 Self-Esteem

Self-esteem was measured using Rosenberg’s Self-Esteem Scale [24]. This scale conceptualizes self-esteem as a single dimension so that the participant can evaluate him or herself comprehensively. This study used a tool of the Rosenberg scale adapted by Jon [25] for the Korean context. The self-esteem scale consists of five positive questions and five negative questions, and the negative questions were scored inversely. Using a 5-point Likert scale, with “strongly disagree” as a 1-point response and “strongly agree” as a 5-point response, with the final score ranging between 10 and 50 points. On this scale, higher points indicated a higher level of self-esteem. In terms of the tool’s reliability, Cronbach’s α was .78 in a previous study [25], and .75 in this research.

#### 2.3.4 Health Promotion Behaviors

Health promotion behaviors are a proactive response to the environment that enables to achieve a higher level of health. Pender [26] has noted that, as an expression of self-actualization tendencies in humans, health promotion behaviors are not related to specific diseases or problems; rather, it is an act aimed at maintaining and promoting an individual’s well-being, self-actualization, and self-fulfillment. This study used a tool adapted and revised by Seo [28] for the Korean context from the Health Promoting Lifestyle Profile (HPLP), which was originally developed by Walker, Sechrist, and Pender [27]. The adapted tool consists of 47 questions: 11 self-actualization questions, 10 health responsibility questions, 12 exercise and nutrition questions, 7 interpersonal relationship questions, and 7 stress management questions. This tool uses a 4-point Likert scale, with “never” being a 1-point response and “regularly” being a 4-point response, with a final score ranging between 47 and 188 points. On this scale, higher points indicated a higher level of health promotion behaviors. In terms of the tool’s reliability, Cronbach’s α was .89 in a previous study [27] and .93 in this research.

### 2.4 Data Collection

Data was collected from college students attending two universities in Korea between November 20 and December 16, 2016. The researcher explained the study’s objectives and the questionnaire items to students enrolled in chapel class after obtaining approval from the minister in charge of the chapel class. Chapel classes were chosen because they comprised all liberal arts classes, regardless of participant major. Questionnaires were disseminated to participants who agreed to participate by providing their consent in writing. It took participants an average of 15 minutes to complete the questionnaire. The questionnaires were then submitted in a collection box.

### 2.5 Ethical Considerations

Appropriate measures were taken to protect participants against coercion or unjust influence during the recruitment or consent process. Only those who voluntarily agreed to participate could fill out the questionnaire; an explanation of the research purposes and the questionnaire was provided. It was made clear to the students that they had the freedom to not participate in this research, that there were no advantages or disadvantages resulting from their participation in this research, and that they could quit at any time even if they had agreed to participate. The participation agreement included a statement about protecting the participants’ anonymity and confidentiality, and explained that participating in this research would have no impact on their grades etcetera.

### 2.6 Data Analysis

The collected data were analyzed using the IBM SPSS 20.0 program. The general characteristics of the participants—as well as their health consciousness, health perception, self-esteem, and health promotion behaviors—were indicated in counts, percentages, means, and standard deviations. The level of health promotion behaviors, depending on participants’ general characteristics, was measured using a t-test and one-way ANOVA. The Pearson’s correlation coefficient was calculated to analyze correlations between variables. In addition, to identify their effect on health promotion behaviors, variables that correlated significantly with health promotion behaviors were entered as independent variables in a multiple regression analysis. Statistical significance was adopted at the level of p<.05.

## 3 RESULTS

### 3.1 Health Promotion Behaviors According to the General Characteristics of Participants

With the exception of four questionnaires, in which responses were invalid, this research analyzed a total of 331 respondents’ questionnaire. The general characteristics of the participants were as follows: the average age was 20.48 years, with 228 participants (68.9%) in their 20s or older; 210 (63.4%) of the participants were women; 2^nd^ grade of university accounted for 204 (61.6%) of the participants; and 202 (61.0%) indicated that they followed no religion. Of the total respondents, 33 (10%) claimed to have experienced a relatively serious disease, while 154 (46.5%) reported that family members, close relatives, or significant others had experienced a serious disease (Table **1**).

Those aged 20 or older had significantly more health promotion behaviors than others (t=2.15, p=.033). An examination of university level as a variable indicated that health promotion behaviors among juniors were significantly higher than those of freshmen (F=2.71, p=.045). There were no significant differences in health promotion behaviors according to sex, religion, university level, or whether college students or their significant others had experienced a relatively serious disease (Table **1**).

### 3.2 Levels of Health Consciousness, Health Perception, Self-Esteem, and Health Promotion Behaviors

On average, the health consciousness score of respondents was 4.15 points, and their health perception score was 2.12 points (both ranging from 1 to 5). On average, the self-esteem score was 3.48 points (range of 1 to 5), and the health promotion behaviors score was 2.57 points (range of 1 to 4). An examination of other categories of health promotion behaviors revealed the following average scores: self-actualization, 3.01 points; health responsibility, 2.20 points; exercise and nutrition, 2.02 points; interpersonal relationship, 3.04 points; and stress management, 2.74 points (Table **2**).

### 3.3 Correlations Between Health Consciousness, Health Perception, Self-Esteem, and Health Promotion Behaviors

Table **3** shows the correlations between health promotion behaviors and other variables. Each variable was tested for normality using the Kolmogorov-Smirnov test and a normal distribution was verified. In addition, the straight-line relationship between the variables was examined and analyzed as Pearson correlations. Health promotion behaviors had positive correlations with health consciousness (r=.421, p<.001), health perception (r=.326, p<.001), and self-esteem (r=.526, p<.001) (Table **3**).

### 3.4 Variables Affecting Health Promotion Behaviors

Multiple regression analysis was used to analyze the effects of health promotion behaviors. Health consciousness, health perception, and self-esteem were used as explanatory variables, and age was set as a control variable. A review of the assumptions of the regression analysis showed that the residuals were normally distributed, and in terms of multicollinearity, tolerances were .81 to .85 and the variation information factor was 1.18 to 1.23. Therefore, multicollinearity was not present. An analysis of the explanatory variables showed that health consciousness, health perception, and self-esteem were statistically significant. A model including these variables showed an explanatory power of 34.9% (F=58.37, p<.001) on the health promotion behaviors of the participants (Table **4**).

## 4 DISCUSSION

The purpose of this research was to identify the extent of college students’ health consciousness, health perception, self-esteem, and health promotion behaviors, and to identify the variables affecting these health promotion behaviors. According to this study’s findings, health promotion behaviors of college students were scored 2.57 points on average. In similar studies, health promotion behaviors in college students were scored 2.31 [29] and 2.67 [30]. Therefore, this research has found an average level of health promotion behaviors overall. When the sub-categories of these behaviors were examined, interpersonal relationship and self-actualization scores were relatively higher than other categories at 3.04 points and 3.01 points, respectively. Exercise and nutrition scores were the lowest at 2.02; this is consistent with previous studies of college students [30-32]. Arguably, the reason that mental health promotion behaviors scores-such as interpersonal relationship and self-actualization-were higher is because the college period is usually during early adulthood, a period in which individuals establish their self-identity and focus on various tasks in preparation for adulthood [33]. However, the finding that the level of exercise and healthy eating was low, and that this is consistent with previous studies, may also be due to the tendency of college students to skip breakfast and to exercise irregularly [34]. Students are also given more freedom upon entering college, and their diet and sleep patterns become irregular [35]. They are more likely to experience lifestyle changes-they leave their home to live in a dormitory, boarding house, or rented room [2]; and there are changes to their support system. Therefore, it may be difficult to maintain health promotion behaviors such as exercise and healthy eating. Additionally, studies show that college students recognize drinking as an important tool in forming interpersonal relationships, and drink frequently in social gatherings with members of clubs, or with junior and senior schoolmates [36]. Students may choose smoking and drinking [37] as a means of reducing stress related to interpersonal relationships (*e.g*., romantic relationships, friendships, or relationships with professors) or stressors related to self-actualization (*e.g*., their future or academic performance). This suggests that the stress of interpersonal relationships or self-actualization can have a negative effect on physical well-being and the promotion of health behaviors. Accordingly, it is necessary to manage mental and physical health in a balanced way, including the use of coping strategies to maintain mental health. Another means of achieving this is through the introduction of intervention measures, such as a change of environment and support systems to accomplish physical health goals, including exercise and good nutrition.

Analysis of the general characteristics of the participants in this study showed that those aged 20 years or older had the highest level of health promotion behaviors, and that juniors displayed a greater degree of these behaviors than freshmen. This is consistent with the results of previous studies, which reported differences in health promotion behaviors depending on the age [22] and university level [10] of respondents. This is likely because freshmen have trouble managing the newfound freedom of college life, relative to the structured environment they experienced during high school [38]. This finding supports those of a report that showed that in the college environment, students gain new responsibilities and experience the stress of needing to adapt to that new environment; consequently, they often choose smoking and drinking as a means of controlling stress [39], and have fewer exercise activities [40]. Accordingly, it is necessary to recognize that students in their freshmen year are particularly vulnerable to health-related problems compared to during other college periods and to highlight the need to develop a comprehensive stress management program that considers the pressures and the specific context of college.

This study found no difference in health promotion behaviors depending on whether college students themselves or their family members, relatives or significant others, had experienced a serious disease. This supports the findings of a previous study, which reported no significant difference in health promotion behaviors if respondents had a family member suffering from a chronic disease [31]. This means that neither the direct nor the indirect experience of the disease can bring about improvements in health promotion behaviors. Considering the studies that identified no correlation between health knowledge and health promotion behaviors [35], it is unlikely that knowledge about or experience of disease influences health promotion behaviors. Rather, it is the degree to which one perceives health as a problem that determines the degree to which knowledge or experience of disease impacts health promotion behaviors. Consequently, it is thought that disease or circumstantial characteristics can lead to various results. This is contradicted by the findings of a study, which argued that the experience of a chronic disease in family members served as an opportunity to realize the importance of health and health-related problems; as a result, this study claimed, those who had someone with chronic disease in their family performed more health promotion behaviors than those without such experience [41]. Given these contradictory findings, additional research examining the impact of direct or indirect disease experience on health promotion behaviors is necessary; if disease experience is proven to have an effect, a strategy linking these findings with health promotion behaviors is required.

A model established based the variables influencing health promotion behaviors found that health consciousness, health perception, and self-esteem were significant. Health consciousness is a concept showing how integrated health interests are in an individual’s daily activities [42]. An individual with a higher level of health consciousness is correspondingly health-oriented with a positive and proactive attitude towards health management overall, including exercise and healthy food. This also seems to contribute to a high level of health promotion behaviors. Accordingly, it seems that a positive attitude towards health has a positive effect on health promotion behaviors. Furthermore, in this research, a higher degree of health perception led to a correspondingly high level of health promotion behaviors; this is consistent with the findings of previous studies [43, 44]. When an individual perceives that his or her health status is good, they perform health promotion behaviors well. This supports the findings of a previous study, which showed that a higher level of perceived health strengthened self-determination and internal motivation, and that this had a direct effect on health behaviors [45]. This implies that the subjective perception of health is an important variable affecting health behaviors. In addition, a higher level of self-esteem resulted in a higher level of health promotion behaviors, which was also consistent with the findings of previous studies [13, 14]. College students with higher self-esteem were shown to have lower stress levels and a positive attitude towards their life, and they thus performed health promotion behaviors appropriately. Conversely, college students with low self-esteem tended to experience depression, anxiety, and higher stress levels, and they therefore experienced more health-related problems [46]. As higher self-esteem equates to a positive attitude towards the practice of health promotion behaviors, it is necessary to develop a program improving self-esteem in college students.

The findings of this research confirm that physical health promotion behaviors among college students may be particularly vulnerable. As college students are in a new and relatively free environment, it is easy for them to neglect their health promotion behaviors in their regular daily activities. Therefore, college students can be particularly vulnerable to health promotion. Given the variables influencing health promotion behaviors in college students-health consciousness, health perception, and self-esteem-this research suggests that it is necessary to perceptions of the importance of health and health management among students, and to develop intervention strategies for the promotion of self-esteem.

## CONCLUSION

This research sought to identify levels of health consciousness, health perception, self-esteem, and health promotion behaviors among college students. To do so, this study examined the variables affecting the promotion of health behaviors. A health promotion behaviors model was established using data from 331 college students, and it demonstrated that health consciousness, health perception, and self-esteem were significant explanatory variables. This model explained 34.9% of variance in health promotion behaviors. Throughout the college period, which is typically during early adulthood, students are exposed to an environment that places their health at risk. Health promotion behaviors are necessary for this age group because they improve health consciousness, health perception, and self-esteem, which in turn enable the maintenance of physical, mental, and social well-being. To achieve this, the development and implementation of a training program are required, so that individuals can integrate healthy behaviors into their daily lives with the understanding that they can protect their own health.

## Figures and Tables

**Table 1:** Differences in variables by general characteristics of participants (N=331).

Characteristics		n (%) or M±SD			Health Promotion Behaviors M±SD	t or F (*p*)
Age (yr)	<20	103	(31.1)	2.50±0.369		2.15
	≥20	228	(68.9)	2.60±0.394		(.033)
		20.48±1.82				
Gender	Male	121	(36.6)	2.66±0.431		-0.26
	Female	210	(63.4)	2.52±0.353		(.796)
Grade	1^a^	96	(29.0)	2.51±0.372		2.71
	2	204	(61.6)	2.57±0.380		(.045)
	3^b^	25	(7.6)	2.76±0.499		a<b
	4	6	(1.8)	2.60±0.190		
Religion	No	202	(61.0)	2.65±0.449		-0.43
	Yes	129	(39.0)	2.52±0.337		(.666)
Experience of severe illness (one's own)	No	298	(90.0)	2.57±0.387		-0.11
	Yes	33	(10.0)	2.56±0.410		(.911)
Experience severe illness (significant others)	No	177	(53.5)	2.59±0.404		-1.16
	Yes	154	(46.5)	2.54±0.370		(.248)

**Table 2:** Description of outcome variables (N=331).

Variables	Range	M±SD
**Health cognition**	*1-5*	4.15±0.570
**Health perception**	*1-5*	2.12±0.250
**Self-esteem**	*1-5*	3.48±0.651
**Health promotion behaviors**	*1-4*	2.57±0.389
*Self-Actualization*		3.01±0.501
*Health Responsibility*		2.20±0.492
*Exercise and Nutrition*		2.02±0.596
*Interpersonal relationships*		3.04±0.496
*Stress Management*		2.74±0.513

**Table 3:** Correlations between outcome variables (N=331).

Variables	**Health Cognition**	**Health Perception**	**Self-Esteem**
	r (*p*)		
****			
**Health perception**	.327(<.001)		
**Self-esteem**	.371(<.001)	.316(<.001)	
**Health promotion behaviors**	.421(<.001)	.326(<.001)	.526(<.001)

**Table 4:** Multiple regression analysis of participants' health promotion behaviors (N=331).

Variables	B	S. E.	β	t (p)	R^2^	F (p)
Intercept	31.80	8.07		3.94 (<.001)	.349	58.37 (<.001)
Health cognition	1.49	.31	.23	4.69 (<.001)		
Health perception	.28	.11	.12	2.55 (.011)		
Self-esteem	1.13	.14	.40	8.14 (<.001)		
